# The relationship of the clinicopathological characteristics and treatment results of post-Chornobyl papillary thyroid microcarcinomas with the latency period and radiation exposure

**DOI:** 10.3389/fendo.2022.1078258

**Published:** 2022-12-14

**Authors:** Tetiana Bogdanova, Serhii Chernyshov, Liudmyla Zurnadzhy, Tatiana I. Rogounovitch, Norisato Mitsutake, Mykola Tronko, Masahiro Ito, Michael Bolgov, Sergii Masiuk, Shunichi Yamashita, Vladimir A. Saenko

**Affiliations:** ^1^ Laboratory of Morphology of Endocrine System, State Institution “VP Komisarenko Institute of Endocrinology and Metabolism of the National Academy of Medical Sciences of Ukraine”, Kyiv, Ukraine; ^2^ Department of Radiation Molecular Epidemiology, Atomic Bomb Disease Institute, Nagasaki University, Nagasaki, Japan; ^3^ Department of Surgery of Endocrine Glands, State Institution “VP Komisarenko Institute of Endocrinology and Metabolism of the National Academy of Medical Sciences of Ukraine”, Kyiv, Ukraine; ^4^ Department of Radiation Medical Sciences, Atomic Bomb Disease Institute, Nagasaki University, Nagasaki, Japan; ^5^ Department of Fundamental and Applied Problems of Endocrinology, State Institution “VP Komisarenko Institute of Endocrinology and Metabolism of the National Academy of Medical Sciences of Ukraine”, Kyiv, Ukraine; ^6^ Department of Diagnostic Pathology, National Hospital Organization Nagasaki Medical Center, Omura, Japan; ^7^ Radiation Protection Laboratory, State Institution “National Research Center of Radiation Medicine of the National Academy of Medical Science of Ukraine”, Kyiv, Ukraine; ^8^ Fukushima Medical University, Fukushima, Japan; ^9^ National Institute of Radiological Sciences, National Institutes for Quantum Science and Technology, Chiba, Japan

**Keywords:** radiogenic papillary thyroid microcarcinoma, Chornobyl, latency period, probability of causation, invasiveness, clinical characteristics, *BRAF^V600E^* mutation

## Abstract

**Introduction:**

A worldwide increase in the incidence of thyroid cancer during the last decades is largely due to papillary thyroid microcarcinomas (MPTCs), which are mostly low-risk tumors. In view of recent clinical recommendations to reduce the extent of surgery for low-risk thyroid cancer, and persisting uncertainty about the impact of radiation history, we set out to address whether clinicopathological characteristics and prognosis of post-Chornobyl MPTCs were changing with regard to: i) the latency period, ii) probability of causation (POC) of a tumor due to radiation, and iii) tumor size.

**Methods:**

Patients (n = 465) aged up to 50 years at diagnosis who lived in April, 1986 in six northern, most radiocontaminated regions of Ukraine were studied.

**Results:**

Latency period was statistically significantly associated with the reduction of POC level, tumor size and the frequency of fully encapsulated MPTCs. In contrast, the frequency of oncocytic changes and the *BRAF^V600E^* mutation increased. Invasive properties and clinical follow-up results did not depend on latency except for a lower frequency of complete remission after postsurgical radioiodine therapy. The POC level was associated with more frequent extrathyroidal extension, and lymphatic/vascular invasion, less frequent oncocytic changes and *BRAF^V600E^*, and did not associate with any clinical indicator. Tumor size was negatively associated with the latency period and *BRAF^V600E^*, and had a statistically significant effect on invasive properties of MPTCs: both the integrative invasiveness score and its components such as lymphatic/vascular invasion, extrathyroidal extension and lymph node metastases increased. The frequency of total thyroidectomy, neck lymph node dissection and radioiodine therapy also increased with the larger tumor size. The duration of the latency period, POC level or tumor size did not associate with the chance of disease recurrence.

**Discussion:**

In summary, we did not observe overall worsening of the clinicopathological features or treatment results of radiogenic MPTCs that could be associated with the latency period or POC level, suggesting that radiation history did not strongly affect those in the analyzed MPTC patients. However, the increase in the invasive properties with tumor size indicates the need for individual risk stratification for each MPTC patient, regardless of radiation history, for treatment decision-making.

## Introduction

The increase in the frequency of detection of papillary thyroid microcarcinomas (MPTCs) sized up to 10 mm during the last decades is well-described in different countries (1–6). This growth is largely due to the progress in ultrasound diagnostics, improvement of fine-needle aspiration biopsy, introduction of screenings, and public awareness of facile thyroid imaging (1–3, 5).

An increased risk of radiation-related MPTCs was reported among victims of the atomic bomb explosions in Japan (7), and in children and adolescents of Ukraine affected by the Chornobyl accident in whom the frequency of MPTCs was growing with time after the accident (8–11). Despite a call not to reduce the extent of surgical treatment of patients with low-risk PTC with a history of radiation exposure in the recent recommendations (12, 13), clinical and histopathological studies that would justify such a warning have not been performed until now.

In our previous work, we compared the clinicopathological characteristics of radiogenic and sporadic MPTCs in the groups of young patients from Ukraine aged up to 30 years at the time of surgery, and did not find evidence that Chornobyl radiation (in this case, internal from ^131^I) affected phenotype of the tumors, increased invasive properties, or worsened prognosis (14).

In the present work, we set out to address whether clinicopathological characteristics and prognosis of MPTCs in patients from Ukraine aged up to 50 years at the surgery who were exposed to internal ^131^I radiation in childhood changed with increasing latency period (i.e., the period between the Chornobyl accident and operation), probability of causation (POC) of a tumor due to radiation, and tumor size.

## Materials and methods

### Patients

Radiogenic MPTCs were from 465 patients aged 8.8 to 50.0 years at the time of diagnosis who were operated on at the State Institution “VP Komisarenko Institute of Endocrinology and Metabolism of the National Academy of Medical Sciences of Ukraine” (IEM), Kyiv during the period from 1992 to 2018 when a significant increase in thyroid cancer incidence after the Chornobyl accident was documented (15–17). Given that the high risk of thyroid cancer was observed in persons who were children and adolescents at the time of Chornobyl accident and lived in the six northern, most radiocontaminated regions of Ukraine (18, 19), we defined inclusion criteria as age up to 18 years in April 1986, living in Kyiv, Chernihiv, Zhytomyr, Rivne, Cherkasy regions or Kyiv city at the time of the Chornobyl accident, non-incidental tumor finding, and the absence of screening history in tumor detection.

The study was conducted according to the guidelines of the Declaration of Helsinki and was approved by the IEM Bioethics Committee (protocols N 22-KE of April 26, 2018, and N 31-KE of February 27, 2020), the Chornobyl Tissue Bank (CTB, project N001-2020), and the Ethics Committee of Nagasaki University (protocol 20130401–7 of July 1, 2021, the latest update). Informed consent was obtained from all patients enrolled in the study or their guardians (for minors).

### Histopathology

Pathological examination of paraffin sections stained with hematoxylin and eosin was performed by two experienced IEM pathologists (TB and LZ). The pathological diagnosis was based on the 4th edition of the WHO histological classification (20). Most of the cases were also reviewed by the international pathology panel of the CTB project (21, 22). The diagnosis of MPTC was confirmed in all analyzed cases, pTNM categories were determined according to the 8th edition of the TNM Classification (23). Tumors were also classified according to the dominant histological structure (papillary, follicular or solid-trabecular), and the presence of oncocytic changes in tumor cells was also evaluated.

As in our previous works (14, 24–26), in addition to the usual clinicopathological features, we used an integrative variable, the “invasiveness score”, which is an unweighted arithmetic sum of each manifestation of multifocality, lymphatic/vascular invasion, any extrathyroidal extension (i.e., minimal to the adipose or connective tissue, or significant to the muscle), pN1 and M1 (distant metastases to the lung were usually detected on postoperative diagnostic imaging), either isolated or in combination with other(s) for each tumor. The invasiveness score determined in this way ranged from “0” (no sign of invasiveness) to “5” (all the above signs present); the actual highest individual invasiveness score observed in this study was “4”.

### Immunohistochemistry

Immunohistochemical (IHC) staining for *BRAF^V600E^* and Ki67 was performed in 95 and 92 MPTCs, respectively, for which the additional paraffin sections with tumor tissue were available. IHC staining for *BRAF^V600E^* expression was performed as previously described (14, 25, 26): a mouse monoclonal antibody to BRAF (V600E mutated protein) (VE1) ab228461 (Abcam) at a 1:100 dilution and a Novolink Polymer Detection System (250T) (Leica RE7140-K) were used to detect the product of IHC reaction. A positive IHC reaction for BRAF^V600E^ was consistent with the presence of the *BRAF^V600E^* mutation (27).

The proliferative activity of tumors was evaluated by IHC using a Ki67 antibody (clone MIB-1; DAKO, Glostrup, Denmark, 1:100 dilution) in a Ventana BenchMark ULTRA instrument. The Ki67 labeling index (Ki67 LI) was determined with the image-analyzing software (CountσCell, Ki67 antigen Semi-Auto Counter, Seiko Tec LTD, Fukuoka, Japan) in a total of approximately 1,000 PTC cells per case (LZ).

### Thyroid dosimetry

^131^І thyroid radiation doses (the absorbed doses in mGy) were calculated for each patient in the Radiation Protection Laboratory of the State Institution “National Research Center for Radiation Medicine of the National Academy of Medical Sciences of Ukraine”, Kyiv using an ecological dosimetric model, which includes the system of ecological iodine transport and biokinetic models of iodine (“TD-CTB”) (28).

### Probability of causation due to radiation

The probability of causation (POC) of a tumor by exposure to a known radiation dose of an individual of a given sex and age after a certain period of latency was determined using the US NIH/NCI Division of Cancer Epidemiology and Genetics’ Interactive RadioEpidemiological Program - Probability of Cancer Causation from Radiation Version 5.7.1 software (29)(https://radiationcalculators.cancer.gov/irep). This software, as mentioned in our previous work (26), uses “Personal Information” such as gender, birth year, diagnosis year and a cancer model (here, the “Thyroid (193)”), and “Dose Exposure Information” such as exposure year (here, 1986), exposure rate (here, the acute), radiation type (here, the electrons E > 15keV as 90% of ^131^I beta-decay has the energy of 606keV), organ dose (here, Constant) and parameter 1 (here, the thyroid dose in cSv; since radiation weighting factor for the beta-particles is 1, the equivalent doses were considered to be numerically equal to the absorbed doses) as input variables. The output are the values of the “Assigned Share (Probability of Causation)” that range from the 1^st^ to the 99^th^ percentile based on 10,000 random-seeded simulations, and the assigned share associated with the expected value of the excess relative risk (ERR). The latter was considered as POC estimate in this study, it is very close to the 50^th^ percentile value. The higher POC value reflects the higher likelihood of cancer development due to radiation exposure.

### Statistical analysis

The Fisher’s exact test, Fisher-Freeman-Halton exact test, and Cochran-Armitage test were used for univariate analysis of categorical data; the Mann-Whitney test was used to compare continuous data between any two groups. Logistic regression models were adjusted for age at operation and sex. Models with very small numbers of outcomes (< 5 per cell) were conducted using Firth’s approach to bias-reducing penalized maximum likelihood fit. To plot estimated probabilities obtained in the logistic regression models (SAS PROC LOGISTIC), the PROC LOESS was used for smoothing. Multivariable linear regression models were applied to continuous dependent variables. The Cox proportional hazard models were used to analyze the effect of latency period on recurrence. Recurrence was defined as a tumor newly detected not earlier than six months after the initial treatment.

Calculations were performed using the version 9.4 of SAS (SAS Institute, Cary, NC, USA) or IBM SPSS Statistics Version 24 software (International Business Machines Corp., Armonk, NY, USA). All tests were two-sided; p < 0.05 was considered indicative of statistical significance.

## Results

### Descriptive characteristics

**Figure 1:**
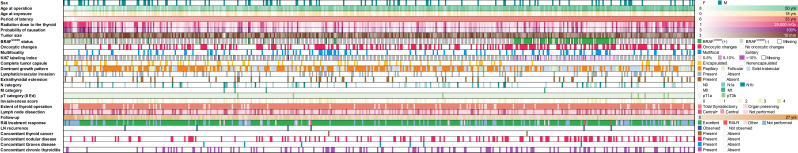
Baseline and clinicopathological profile of 465 radiogenic papillary thyroid microcarcinomas in the study.

Radiogenic MPTCs detected by ultrasound and assessed by FNA/cytology as malignant or suspicious for malignancy accounted for 465 cases in the current analysis. All data collected for or generated during the study are presented in **Figure 1**.

The female/male ratio was 3.7:1, the median age of patients at the time of surgery was 35.0 (8.8-50.0, range) years and 9.2 (0.1-18.3, range) years at the time of the Chornobyl accident, latency period was 26.2 (6.2-32.7, range) years, and tumor size was 7 (3-10, range) mm. MPTCs more commonly had the dominant papillary or solid-trabecular patterns (48.8% and 30.6%, respectively), the median Ki67 LI was 4.6% (0.4-18.7%, range). In 67.4% MPTCs were BRAF^V600E^-positive, oncocytic changes were noted in 43.2%, multifocal growth in 22.2%, lymphatic-vascular invasion in 24.9%, extrathyroidal extension in 13.8%, metastases to the regional lymph nodes in 19.4%, and distant metastases to the lung in 0.9% (**Table 1**).

**Table 1:** Descriptive characteristics of the 465 radiogenic papillary thyroid microcarcinomas in the study and their associations with patient age and sex.

	number or value	Age		Sex	
Parameters	(% or IQR or SD)	OR, b or HR (95%CI)	p-value	OR, b or HR (95%CI)	p-value
**Sex**, F/M; %M; F:M ratio (ref=F)	367/98; 21.1%; 3.7:1	0.974 (0.947-1.002)	0.073	NA^a^	NA
**Age at operation**, years	35.0 (29.5-39.9)	NA	NA	-10.351 (-25.003-4.301)	0.166
**Age at exposure**, years	9.2 (4.7-13.8)	**0.374 (0.341-0.407)**	**8.51E-76**	-0.221 (-1.111-0.669)	0.626
**Latency period**, years	26.2 (26.2 (22.6-29.1)	**19.114 (17.311-20.918)**	**1.79E-68**	**-48.616 (-95.575-1.656)**	**0.042**
**Radiation dose to the thyroid**, mGy	46.8 (27.7-113.2)	**-0.043 (-0.051- -0.35)**	**1.01E-22**	0.140 (-0.029-0.310)	0.105
**Probability of causation**, %	19.1 (8.6-46.9)	**-0.126 (-0.142- -0.110)**	**1.37E-43**	0.171 (-0.199-0.542)	0.364
≤ 25%	261 (56.1%)	**1.200 (1.156-1.246)**	**1.62E-21**	0.855 (0.546-1.337)	0.491
> 25 – 50%	101 (21.7%)	**0.971 (0.944-0.999)**	**0.041**	0.905 (0.522-1.569)	0.723
> 50 – 75%	67 (14.4%)	**0.876 (0.845-0.909)**	**1.71E-12**	0.987 (0.523-1.866)	0.969
> 75 – 100%	36 (7.7%)	**0.867 (0.829-0.907)**	**5.49E-10**	1.994 (0.959-4.147)	0.065
**Tumor size**, mm	7.0 (6-9)	-0.008 (-0.050-0.34)	0.709	0.158 (-0.632-0.948)	0.695
lesser or equal median	242 (52.0)	0.999 (0.976-1.022)	0.930	1.053 (0.674-1.645)	0.820
greater than median	223 (48.0)	1.001 (0.978-1.025)	0.930	0.950 (0.608-1.484)	0.820
**Full tumor capsule**	80 (17.2)	**0.969 (0.940-0.999)**	**0.044**	0.924 (0.507-1.683)	0.796
**Dominant growth pattern**		1.016 (0.993-1.039)	0.175	1.798 (0.522-1.220)	0.297
papillary	227 (48.8)	0.991 (0.967-1.014)	0.438	1.118 (0.716-1.746)	0.623
follicular	96 (20.6)	0.981 (0.953-1.009)	0.185	1.428 (0.846-2.408)	0.182
solid-trabecular	142 (30.6)	**1.028 (1.001-1.056)**	**0.045**	0.639 (0.382-1.070)	0.089
**Ki-67 labeling index**, n=92	4.6 (2.9-7.2)	-0.003 (-0.021-0.015)	0.781	-0.199 (-0.605-0.207)	0.332
0 – 5%	52 (56.5%)	1.023 (0.982-1.065)	0.280	1.396 (0.538-3.626)	0.493
>5 – 10%	34 (37.0%)	0.983 (0.943-1.024)	0.413	0.808 (0.303-2.151)	0.669
>10%	6 (6.5%)	0.978 (0.904-1.057)	0.569	0.548 (0.061-4.942)	0.592
***BRAF^V600E^*-positive**, n=95	64 (67.4%)	**1.119 (1.063-1.177)**	**1.50E-05**	**0.353 (0.138-0.902)**	**0.03**
**Oncocytic changes**	201 (43.2%)	**1.054 (1.027-1.081)**	**7.10E-05**	0.836 (0.531-1.317)	0.441
**Multifocality**	103 (22.2%)	1.027 (0.997-1.058)	0.081	0.877 (0.507-1.519)	0.640
**Lymphatic/vascular invasion**	116 (24.9%)	0.983 (0.957-1.010)	0.213	1.535 (0.940-2.506)	0.086
**Extrathyroidal extension (any)**	64 (13.8%)	0.993 (0.960-1.027)	0.674	1.721 (0.954-3.105)	0.071
**T category**					
pT1a	458 (98.5%)	0.972 (0.876-1.079)	0.597	0.663 (0.127-3.470)	0.626
pT3b	7 (1.5%)	1.028 (0.927-1.141)	0.597	1.508 (0.288-7.895)	0.626
**N category (N1)**	90 (19.4%)	1.001 (0.971-1.032)	0.944	**1.710 (1.013-2.885)**	**0.045**
N1a	56 (12.0%)	0.983 (0.948-1.018)	0.334	1.437 (0.759-2.721)	0.266
N1b	34 (7.3%)	1.033 (0.984-1.085)	0.190	1.891 (0.888-4.028)	0.099
**M category (M1)**	4 (0.9%)	0.942 (0.840-1.055)	0.299	3.802 (0.529-27.340)	0.185
**Invasiveness score**	1 (0-1)	1.001 (0.979-1.024)	0.900	1.451 (0.961-2.190)	0.077
0	224 (48.2%)	0.995 (0.972-1.019)	0.673	0.847 (0.541-1.324)	0.466
1	141 (30.3%)	1.012 (0.986-1.039)	0.383	0.693 (0.416-1.154)	0.159
2	69 (14.8%)	0.995 (0.962-1.028)	0.750	1.271 (0.698-2.316)	0.433
3	26 (5.6%)	0.988 (0.940-1.039)	0.641	3.518 (1.571-7.879)	0.002
4	5 (1.1%)	1.018 (0.903-1.148)	0.773	0.936 (0.103-8.467)	0.953
5	0	NA	NA	NA	NA
**Concomitant thyroid cancer**	2 (0.4%)	1.133 (0.928-1.384)	0.219	0.742 (0.035-15.798)	0.848
**Concomitant nodular disease**	120 (25.8%)	**1.059 (1.027-1.091)**	**2.34E-04**	0.585 (0.334-1.023)	0.060
**Concomitant Graves’ disease**	7 (1.5%)	1.047 (0.939-1.167)	0.412	0.244 (0.014-4.370)	0.338
**Chronic thyroiditis**	121 (26.0%)	**1.029 (1.000-1.058)**	**0.049**	**0.262 (0.131-0.523)**	**1.46E-04**
**Thyroid surgery**					
total thyroidectomy	405 (87.1%)	1.026 (0.991-1.061)	0.143	0.698 (0.375-1.300)	0.257
organ-preserving operation	60 (12.9%)	0.975 (0.942-1.009)	0.143	1.432 (0.769-2.666)	0.257
**LN dissection performed**	192 (41.3%)	1.000 (0.977-1.025)	0.973	1.411 (0.901-2.209)	0.132
level ≥ 6	132 (28.4%)	1.016 (0.989-1.043)	0.254	0.889 (0.538-1.469)	0.646
level 1 – 5	60 (12.9%)	0.975 (0.942-1.008)	0.138	**2.294 (1.277-4.120)**	**0.005**
**RIT performed**	354 (76.1%)	1.024 (0.996-1.052)	0.090	0.642 (0.391-1.054)	0.079
**RIT cycles**	1 (1-1)	0.997 (0.973-1.022)	0.809	0.908 (0.575-1.434)	0.679
**Cumulative RI activity**, MBq	3964 (2775-4360)	0.011 (-0.001-0.023)	0.066	0.194 (-0.031-0.418)	0.091
**RIT response**, n=354		0.953 (0.803-1.018)	0.156	**0.279 (0.113-0.690)**	**0.006**
RAI-R recurrence *vs* other	3 (0.8%)	1.174 (0.963-1.425)	0.103	7.155 (0.921-55.565)	0.060
excellent *vs* other	333 (94.1)	0.954 (0.894-1.019)	0.165	**0.285 (0.117-0.697)**	**0.006**
**Follow-up**, years	5.2 (2.2-9.1)	**-0.095 (-0.115- -0.074)**	**8.25E-19**	0.098 (-0.313-0.509)	0.639
**Recurrence**	6 (1.3%)	1.016 (0.913-1.131)	0.767	3.646 (0.732-18.161)	0.114
**Time to recurrence**, yrs	1.2 (1.1-1.6)	-0.001 (-0.529-0.528)	0.998	3.496 (-5.047-12.020)	0.320
**Recurrent metastases**, n=6					
Dominant growth pattern					
papillary	5 (83.3%)	1.134 (0.870-1.478)	0.352	0.238 (0.004-15.012)	0.497
follicular	1 (16.7%)	0.882 (0.677-1.149)	0.352	4.199 (0.067-264.725)	0.497
solid-trabecular	0	NA	NA	NA	NA
Ki67 labeling index, n=3	1.2 (1.0-1.9)	0.069 (-0.470-0.608)	0.351	-0.450 (-19.157-18.257)	0.811
*BRAF^V600E^*-positive, n=3	2 (66.7%)	1.245 (0.809-1.915)	0.319	2.999 (0.015-605.350)	0.685
Oncocytic changes	3 (50.0%)	1.108 (0.901-1.363)	0.332	1.360 (0.013-9.816)	0.545
Cystic changes	5 (83.3%)	1.049 (0.846-1.302)	0.660	4.199 (0.067-264.725)	0.497
RIT recurrence response					
RAI-R recurrence *vs* other	3 (50.0%)	1.202 (0.904-1.599)	0.206	2.777 (0.102-75.720)	0.545
excellent *vs* other	3 (50.0%)	0.832 (0.626-1.106)	0.206	0.360 (0.013-9.816)	0.545

a Not available.

Numbers in bold indicate statistical significance.

Total thyroidectomy was performed in 87.1%, and postoperative radioiodine therapy (RIT) in 76.1% of cases. The median follow-up period was 5.2 (0.1-27.0, range) years, during which the recurrences, all to the regional lymph nodes, were detected and operated on in 1.3% of patients.

As seen in **Table 1**, some characteristics were associated with patient age and sex. While age at exposure, period of latency, radiation thyroid dose, POC and follow-up duration expectedly correlated with age at operation due to their nature or definition (age at operation is highly correlated with age at exposure and latency period, while the two latter parameters do not correlate), the reasons for associations for other (such as e.g. tumor capsule) were not obvious or easily predictable. Therefore, in further analyses we adjusted multivariable regression models for age at operation and sex whenever reasonable.

### Effect of the latency period

**Figure 2:**
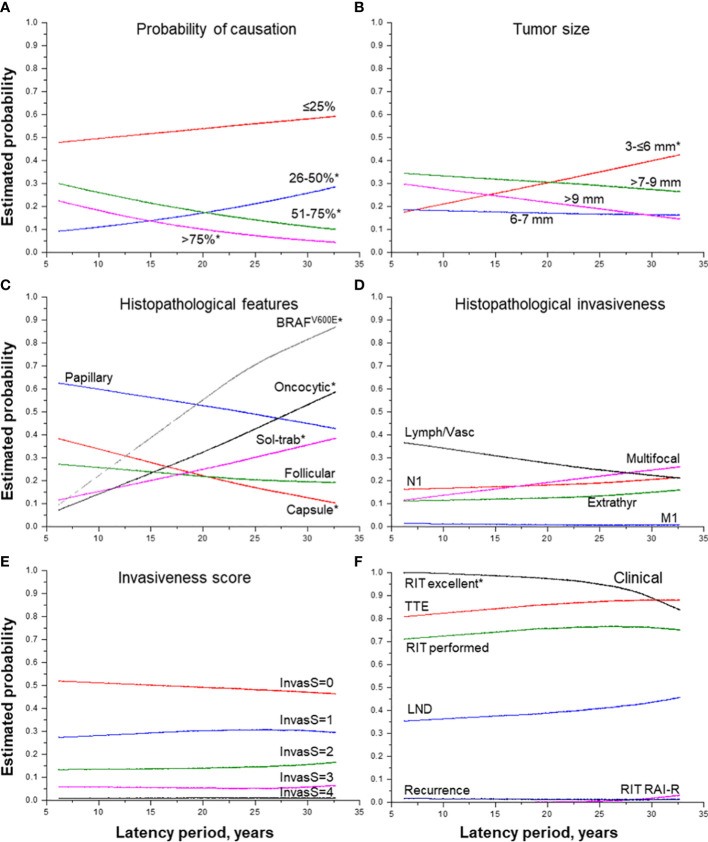
Effect of the period of latency on MPTC characteristics. **(A)** probability of causation (by 25%), **(B)** tumor size (by groups), **(C)** histopathological features, **(D)** histopathological features of tumor invasiveness, **(E)** the integrative invasiveness score, and **(F)** clinical parameters. RIT, radioiodine therapy; TTE, total thyroidectomy; LND, lymph node dissection; RAI-R, radioiodine-refractory. * indicate statistical significance, p < 0.05.

As shown in **Table 2**, the increasing latency period expectedly decreased POC (b = -0.029, p = 0.044) due to changes in its components, namely the increase in patient age at the time of surgery (b =8.648, p = 1.09E-69) and reduction of thyroid dose (b = -0.027, p = 0.001). The estimated probability of detecting MPTC with POC of 51-75% 30 years after the Chornobyl accident was about 15%, and that of > 75% was about 5% only (**Figure 2A**).

**Table 2:** Associations of radiogenic MPTCs with the latency period, probability of causation due to radiation, and tumor size.

	Latency period		Probability of causation		Tumor size	
Parameters	OR, b or HR (95%CI)^a^	p-value	OR, b or HR (95%CI)^a^	p-value	OR, b or HR (95%CI)^a^	p-value
**Sex**, F/M; %M; F:M ratio (ref=F)	**0.956 (0.918-0.995)** ^b^	**0.027**	1.006 (0.998-1.015)^b^	0.148	1.026 (0.903-1.164)^b^	0.697
**Age at operation**, years	**8.648 (7.843-9.454)** ^c^	**1.09E-69**	**-0.187 (-0.209- -0.164)** ^c^	**8.22E-48**	-0.938 (-4.333-2.458)^c^	0.588
**Age at exposure**, years	0.033 (-0.035-0.102)^c^	0.339	**-0.158 (-0.171- -0.146)** ^c^	**5.98E-84**	0.189 (-0.016-0.395)^c^	0.071
**Latency period**, years	NA^d^	NA	**-0.028 (-0.048- - 0.009)**	**0.004**	**-14.219 (-25.025-3.412)**	**0.010**
**Radiation dose to the thyroid**, mGy	**-0.027 (-0.044- -0.011)**	**0.001**	**13.575 (9.464-17.658)**	**2.21E-10**	-0.004 (-0.043-0.036)	0.856
**Probability of causation**, %	**-0.029 (-0.058- -0.001)** ^e^	**0.044**	NA	NA	-0.053 (-0.139-0.032)^e^	0.221
≤ 25%	1.016 (0.982-1.052)^e^	0.359	NA	NA	1.104 (0.994-1.226)^e^	0.064
> 25 – 50%	**1.053 (1.006-1.102)** ^e^	**0.027**	NA	NA	0.968 (0.854-1.097)^e^	0.608
> 50 – 75%	**0.950 (0.907-0.994)** ^e^	**0.027**	NA	NA	**0.839 (0.722-0.976)**^e^	**0.023**
> 75 – 100%	**0.939 (0.886-0.995)** ^e^	**0.032**	NA	NA	1.035 (0.854-1.526)^e^	0.724
**Tumor size**, mm	**-0.072 (-0.133- -0.012)**	**0.019**	-0.009 (-0.022-0.004)	0.177	NA	NA
lesser or equal median	**1.036 (1.001-1.073)**	**0.043**	**1.009 (1.002-1.017)**	**0.017**	NA	NA
greater than median	**0.965 (0.932-0.999)**	**0.043**	**0.991 (0.983-0.998)**	**0.017**	NA	NA
**Full tumor capsule**	**0.934 (0.895-0.975)**	**0.002**	1.001 (0.991-1.011)	0.862	0.949 (0.827-1.090)	0.460
**Dominant growth pattern**	1.044 (0.996-1.094)^f^	0.076	1.004 (0.998-1.011)^f^	0.211	**0.900 (0.816-0.992)**^f^	**0.034**
papillary	0.970 (0.937-1.004)	0.083	**0.993 (0.985-1.000)**	**0.049**	**1.112 (1.002-1.234)**	**0.045**
follicular	0.988 (0.948-1.030)	0.565	**1.010 (1.002-1.019)**	**0.018**	0.977 (0.860-1.111)	0.724
solid-trabecular	**1.051 (1.010-1.094)**	**0.015**	1.000 (0.992-1.008)	0.970	0.897 (0.801-1.005)	0.062
**Ki-67 labeling index**, n=92	0.019 (-0.014-0.053)	0.259	0.038 (-0.071-0.146)	0.492	0.015 (-0.390-0.420)	0.942
0 – 5%	0.947 (0.869-1.030)	0.205	0.992 (0.979-1.006)	0.261	0.958 (0.740-1.239)	0.743
> 5 – 10%	1.028 (0.945-1.119)	0.515	1.006 (0.992-1.020)	0.396	0.996 (0.766-1.296)	0.979
> 10%	1.119 (0.910-1.377)	0.286	1.008 (0.982-1.035)	0.549	1.212 (0.719-2.041)	0.470
***BRAF^V600E^*-positive**, n=95	**1.138 (1.035-1.252)**	**0.008**	**0.976 (0.962-0.991)**	**0.002**	**0.678 (0.486-0.946)**	**0.022**
**Oncocytic changes**	**1.092 (1.050-1.135)**	**9.00E-06**	**0.990 (0.982-0.997)**	**0.007**	0.969 (0.872-1.077)	0.561
**Multifocality**	1.033 (0.989-1.079)	0.148	0.995 (0.986-1.004)	0.286	0.944 (0.833-1.070)	0.366
**Lymphatic/vascular invasion**	0.975 (0.938-1.013)	0.196	**1.009 (1.000-1.017)**	**0.041**	**1.201 (1.063-1.358)**	**0.003**
**Extrathyroidal extension (any)**	1.026 (0.975-1.080)	0.330	**1.010 (1.000-1.020)**	**0.046**	**1.214 (1.040-1.417)**	**0.014**
**T category**						
pT1a	**0.768 (0.599-0.983)**	**0.036**	0.980 (0.955-1.005)	0.122	0.916 (0.597-1.405)	0.686
pT3b	**1.303 (1.017-1.669)**	**0.036**	1.021 (0.995-1.047)	0.122	1.092 (0.712-1.675)	0.686
**N category (N1)**	1.020 (0.976-1.067)	0.378	1.004 (0.995-1.013)	0.420	**1.208 (1.056-1.382)**	**0.006**
N1a	1.027 (0.973-1.085)	0.326	1.005 (0.995-1.016)	0.320	**1.258 (1.067-1.485)**	**0.006**
N1b	1.003 (0.937-1.073)	0.935	1.000 (0.986-1.014)	0.982	1.085 (0.887-1.326)	0.429
**M category (M1)**	1.010 (0.851-1.198)	0.913	1.024 (0.989-1.059)	0.178	0.926 (0.525-1.633)	0.791
**Invasiveness score**	1.020 (0.973-1.068)^f^	0.410	1.006 (0.999-1.013)^f^	0.099	**1.152 (1.044-1.269)**^f^	**0.005**
0	0.990 (0.957-1.025)	0.581	0.996 (0.988-1.003)	0.237	**0.884 (0.796-0.981)**	**0.021**
1	0.998 (0.961-1.037)	0.934	0.998 (0.990-1.006)	0.603	1.001 (0.895-1.121)	0.982
2	1.015 (0.966-1.066)	0.561	1.009 (0.999-1.019)	0.066	**1.164 (1.004-1.349)**	**0.044**
3	1.022 (0.948-1.101)	0.577	1.006 (0.991-1.021)	0.440	1.206 (0.954-1.524)	0.118
4	0.997 (0.842-1.180)	0.972	0.999 (0.964-1.035)	0.953	1.200 (0.718-2.006)	0.487
5	NA	NA	NA	NA	NA	NA
**Concomitant thyroid cancer**	1.029 (0.829-1.276)	0.798	0.969 (0.901-1.043)	0.399	3.234 (0.950-11.004)	0.060
**Concomitant nodular disease**	**1.089 (1.040-1.141)**	**3.30E-04**	**0.990 (0.981-0.999)**	**0.033**	0.691 (0.852-1.084)	0.519
**Concomitant Graves’ disease**	1.010 (0.866-1.179)	0.896	0.990 (0.956-1.024)	0.549	0.741 (0.467-1.176)	0.203
**Chronic thyroiditis**	**1.068 (1.021-1.118)**	**0.004**	0.998 (0.989-1.006)	0.622	1.017 (0.902-1.146)	0.785
**Thyroid surgery**						
total thyroidectomy	1.015 (0.966-1.066)	0.556	1.000 (0.990-1.011)	0.934	**1.225 (1.044-1.439)**	**0.013**
organ-preserving operation	0.985 (0.938-1.035)	0.556	1.000 (0.989-1.011)	0.934	**0.816 (0.695-0.958)**	**0.013**
**LN dissection performed**	1.022 (0.986-1.058)	0.235	1.002 (0.994-1.009)	0.639	**1.225 (1.044-1.439)**	**0.013**
level ≥ 6	**1.071 (1.027-1.117)**	**0.001**	1.004 (0.996-1.012)	0.277	1.068 (0.952-1.199)	0.259
level 1 – 5	**0.938 (0.894-0.984)**	**0.009**	0.996 (0.984-1.007)	0.438	1.171 (0.999-1.372)	0.051
**RIT performed**	1.002 (0.963-1.043)	0.915	0.993 (0.985-1.001)	0.094	**1.144 (1.011-1.295)**	**0.033**
**RIT cycles**	0.984 (0.950-1.019)^f^	0.370	0.999 (0.992-1.007)^f^	0.866	**1.156 (1.038-1.287)**^f^	**0.008**
**Cumulative RI activity**, MBq	**0.034 (0.017-0.050)**	**6.60E-05**	-0.020 (0.074-0.0340)	0.462	0.026 (-0.024-0.076)	0.312
**RIT response**, n=354	**0.813 (0.692-0.954)**^f^	0.011	0.999 (0.982-1.017)^f^	0.933	0.973 (0.756-1.253)^f^	0.834
RAI-R recurrence *vs* other	1.305 (0.876-1.945)	0.191	0.919 (0.986-1.073)	0.285	0.826 (0.394-1.736)	0.615
excellent *vs* other	**0.836 (0.737-0.949)**	**0.006**	0.999 (0.981-1.017)	0.900	0.954 (0.740-1.230)	0.714
**Follow-up**, years	**-0.202 (-0.228- -0.176)**	**1.51E-43**	0.028 (-0.073-0.129)	0.586	0.062 (-0.025-0.150)	0.160
**Recurrence**	1.070 (0.904-1.266)^g^	0.431	0.993 (0.959-1.027)^g^	0.677	1.348 (0.814-2.232)^g^	0.246
**Time to recurrence**, years	0.016 (-1.915-1.947)	0.975	-0.447 (-3.630-2.736)	0.717	-1.331 (-5.489-2.827)	0.302
**Recurrent metastases**, n=6						
Dominant growth pattern						
papillary	1.217 (0.587-2.523)	0.597	1.000 (0.901-1.110)	1.000	0.405 (0.020-8.017)	0.553
follicular	0.822 (0.396-1.703)	0.597	1.000 (0.901-1.110)	1.000	2.467 (0.125-48.795)	0.553
solid-trabecular	NA	NA	NA	NA	NA	NA
Ki67 labeling index, n=3	NA	NA	-0.447 (-3.630-2.736)	0.717	NA	NA
*BRAF^V600E^*-positive, n=3	1.394 (0.485-4.010)	0.537	1.000 (0.915-1.093)	1.000	0.361 (0.015-8.634)	0.529
Oncocytic changes	1.349 (0.786-2.313)	0.277	1.004 (0.929-1.086)	0.915	1.342 (0.098-18.315)	0.825
Cystic changes	1.045 (0.681-1.603)	0.841	1.000 (0.901-1.110)	1.000	1.467 (0.096-22.347)	0.783
RIT recurrence response						
RAI-R recurrence *vs* other	1.191 (0.456-3.111)	0.721	0.928 (0.813-1.058)	0.263	0.842 (0.054-13.147)	0.903
excellent *vs* other	0.839 (0.321-2.192)	0.721	1.078 (0.945-1.230)	0.263	1.187 (0.076-18.531)	0.903

a Adjusted for age at operation and sex unless otherwise specified.

b Adjusted for age at operation.

c Adjusted for sex.

d Not available.

e Non-adjusted.

f Polytomous logistic regression.

g Cox regression.

Numbers in bold indicate statistical significance.

The longer latency period was also associated with a decrease in tumor size (b = -0.072, p = 0.019) paralleled by a significant increase in the probability of detecting the smallest MPTCs sized from 3 to 6 mm (**Figure 2B**), with a higher frequency of tumors with the dominant solid-trabecular structure (OR =1.051, p = 0.015), oncocytic changes (OR = 1.092, p = 9.00E-06), *BRAF^V600E^-positivity* (OR = 1.138, p = 0.008), as well as with a lower frequency of fully encapsulated tumors (OR = 0.934, p = 0.002) (**Figure 2C**). At the same time, invasive properties did not significantly change with tumor latency (see **Table 2** and **Figures 2D–F**) except for a higher frequency of pT3b tumors (OR = 1.303, p = 0.036). Among clinical characteristics, the declining frequency of excellent response to RIT (OR = 0.836, p = 0.006, and **Figure 2F**) was only noted. This was accompanied by a more frequent, but not statistically significant increase in the frequency of radioiodine-refractory (RAI-R) response (OR = 1.305, p = 0.191) and elevated chance of recurrence (HR = 1.070, p = 0.431).

A more detailed examination of MPTCs with morphological signs of extrathyroidal extension to the muscle (the pT3b category) established that such tumors, despite a small number of cases, were statistically significantly associated, in addition to a longer latency period, with the POC level from 50 to 75% (OR = 1.665, p = 0.049), higher frequencies of lymph node metastases (OR = 10.807, p = 0.005), and invasiveness score “3” (OR = 14.813, p = 0.001) (**Supplementary Table 1**). However, there were no recurrences after a median follow-up of 3.6 years.

### Effect of probability of causation due to radiation

**Figure 3:**
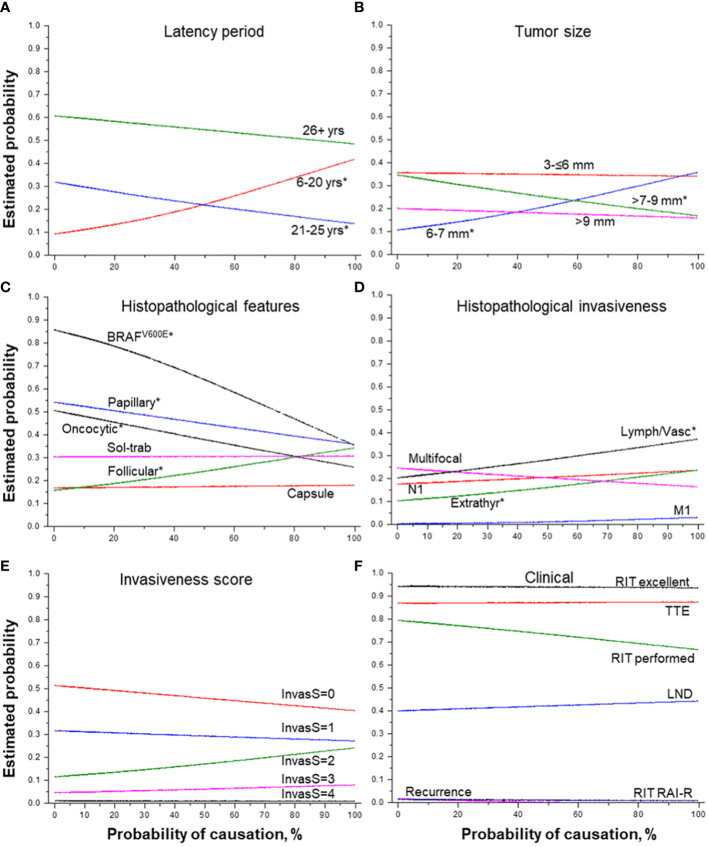
Effect of the probability of causation of a tumor due to radiation on MPTC characteristics. **(A)** latency period (by groups), **(B)** tumor size (by groups), **(C)** histopathological features, **(D)** histopathological features of tumor invasiveness, **(E)** the integrative invasiveness score, and **(F)** clinical parameters. RIT, radioiodine therapy; TTE, total thyroidectomy; LND, lymph node dissection; RAI-R, radioiodine-refractory. * indicate statistical significance, p < 0.05.

The increasing POC (**Table 2**) was naturally associated with a younger age of patients at the time of the Chornobyl accident (b = -0.158, p = 5.98E-04) and at operation (b = -0.187, p = 8.22E-48), the shorter latency (b = -0.028, p = 2.21E-10), and significantly higher ^131^I thyroid dose (b = 13.575, p = 2.21E-10). The probability of detecting MPTCs with the highest POC was significantly increasing for the tumors with the latency period from 6 to 20 years and, conversely, it was decreasing for the tumors with the latency period of 21-25 years (**Figure 3A**). The probability of detecting tumors after the longest latency (26+ years) was slightly declining with POC, yet it remained the highest (50-60%) across POC values.

The higher POC level was positively associated with tumor size slightly smaller than the median (6-7 mm), while the probability of detecting MPTCs of larger size (>7-9 mm) was significantly decreasing (**Figure 3B**). The increasing POC was also associated with the higher frequency of MPTCs with the follicular dominant growth pattern (OR = 1.010, p = 0.018), and less frequent papillary structures (OR = 0.993, p = 0.049), oncocytic changes (OR = 0.990, p = 0.007) and BRAF^V600E^ (OR = 0.976, p = 0.002) (see **Table 2** and **Figure 3C**). The higher POC level was also associated with more frequent lymphatic/vascular invasion (OR = 1.009, p = 0.041) and extrathyroidal extension (OR = 1.010, p = 0.046), but not with the integrative invasiveness score or any clinical characteristic (see **Table 2** and **Figures 3D–F**).

### Effect of tumor size

**Figure 4:**
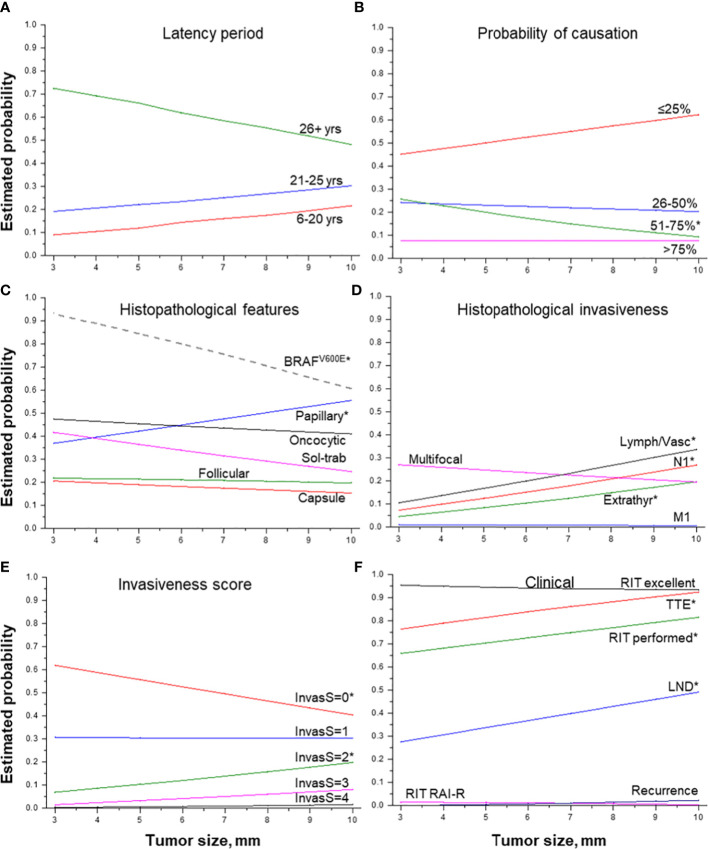
Effect of tumor size on MPTC characteristics. **(A)** latency period (by groups), **(B)** probability of causation (by 25%), **(C)** histopathological features, **(D)** histopathological features of tumor invasiveness, **(E)** the integrative invasiveness score, and **(F)** clinical parameters. RIT, radioiodine therapy; TTE, total thyroidectomy; LND, lymph node dissection; RAI-R, radioiodine-refractory. * indicate statistical significance, p < 0.05.

MPTC size (**Table 2** and **Figures 4A–F**) was negatively associated with the longer latency period (b = -14.219, p = 0.010), especially for latency of more than 26 years, and with POC level from 51% to 75% (OR = 0.839, p = 0.023). MPTCs of larger size more frequently had dominant papillary structure (OR = 1.112, p = 0.045), but tumors with the *BRAF^V600E^* mutation among them were found significantly less frequently (OR = 0.678, p = 0.022). In contrast, an increase in tumor size was statistically significantly associated with higher MPTC invasiveness: both the integrative invasiveness score (OR = 1.152, p = 0.005) and its components such as lymphatic/vascular invasion (OR = 1.201, p = 0.003), extrathyroidal extension (OR = 1.214, p = 0.014), lymph nodes metastases (OR = 1.208, p = 0.006). Total thyroidectomy (OR = 1.325, p = 0.013), lymph node dissection (OR = 1.325, p = 0.013), and postoperative RIT (OR = 1.144, p = 0.033) were also performed more frequently for tumors of increasing size. However, the larger size of MPTCs did not significantly affect the chance of disease recurrence (see **Table 3** and **Figure 4F**).

**Table 3:** Effects of thyroid dose and tumor size on extrathyroidal extension.

	OR (95% CI)	p-value	AIC^a^		OR (95% CI)	p-value	AIC
***POC and its components***				***Tumor size***			
*Non-adjusted*					*Non-adjusted*		
POC	**1.010 (1.000-1.020)**	0.046	372.749	Tumor size, mm	**1.215 (1.042-1.417)**	**0.013**	370.264
Sex	1.721 (0.954-3.105)	0.071	373.517				
Age at exposure, years	0.966 (0.919-1.016)	0.183	374.819				
Latency period, years	1.019 (0.968-1.073)	0.473	376.073				
Age at operation, years	0.993 (0.960-1.027)	0.674	376.425				
Thyroid dose, mGy (log)	**1.266 (1.013-1.583)**	**0.038**	372.406				
	*Model 1A*		374.353		*Model 1B*		369.399
Sex	1.701 (0.930-3.110)	0.085		Sex	1.779 (0.972-3.256)	0.062	
Age at exposure, years	0.992 (0.933-1.055)	0.795		Age at exposure, years	0.961 (0.913-1.011)	0.122	
Latency, years	1.033 (0.981-1.088)	0.214		Latency period, years	1.034 (0.981-2.091)	0.21	
Thyroid dose, mGy (log)	1.248 (0.942-1.653)	0.123		Tumor size, mm	**1.239 (1.060-1.450)**	**0.007**	
	*Model 2A*		373.399		*Model 2B*		371.243
Sex	1.636 (0.899-2.978)	0.107		Sex	1.699 (0.934-3.090)	0.082	
Age at operation, years	1.016 (0.977-1.057)	0.424		Age at operation, years	0.996 (0.962-1.031)	0.829	
Thyroid dose, mGy (log)	**1.313 (1.005-1.714)**	**0.046**		Tumor size, mm	**1.214 (1.040-1.417)**	**0.014**	
***Models combining POC or its components and tumor size***							
	*Model 3A*		369.003		*Model 3B*		
Sex	1.705 (0.926-3.138)	0.086			*Could not be fitted*		
Age at exposure, years	0.988 (0.928-1.052)	0.703		Sex			
Latency period, years	1.042 (0.988-1.099)	0.13		Age at operation, years			
Thyroid dose, mGy (log)	1.251 (0.938-1.668)	0.128		Thyroid dose, mGy (log)			
Tumor size, mm	**1.240 (1.059-1.452)**	**0.008**		Tumor size, mm			
						

^a^Akaike information criterion.Numbers in bold indicate statistical significance.

### Relationships between tumor invasive features, radiation exposure and tumor size

In the course of this work, we found, on the one hand, that lymphatic/vascular invasion and extrathyroidal extension were positively associated with both POC and tumor size (see **Table 2**). On the other hand, the probability of detecting tumors larger than the median size (7 mm in the current study) significantly decreased, and tumor size in general tended to decrease with increasing POC (see **Table 2**). That is, a certain controversy could be seen. In order to discriminate between the radiation and tumor size effects on tumor invasive features, we performed additional analyses, considering POC components (gender, age at the time of exposure, latency period, age at the time of surgery, ^131^I thyroid dose) and tumor size as possible explanatory variables for the lymphatic/vascular invasion and extrathyroidal extension outcomes.

For this purpose, we determined non-adjusted effects (in terms of ORs) of all these parameters and also their effects in multivariable models (avoiding multicollinearity). **Table 4** presents the results for lymphatic/vascular invasion. Non-adjusted effects of thyroid dose and of tumor size were statistically significant, OR = 1.270 (p = 0.012) and OR = 1.202 (p = 0.003), respectively. Then we tested models combining different variables, and observed that that radiation dose and tumor size retained statistical significance in all of them (Models 1A, 1B, 2A and 2B). Finally, in the models which included both radiation dose and tumor size (Models 3A and 3B), both of these variables had statistically significant effects. Furthermore, effect sizes in these models (i.e., OR~1.3 for radiation dose and OR~1.2 for tumor size) did not markedly change (<10% as a rule of thumb) as compared to non-adjusted models. Based on these observations, it is plausible to conclude that radiation dose and MPTC size contributed to the risk of lymphatic/vascular invasion independently.

**Table 4:** Effects of thyroid dose and tumor size on lymphovascular invasion.

	OR (95% CI)	p-value	AIC^a^		OR (95% CI)	p-value	AIC
***POC or its components***				***Tumor size***			
*Non-adjusted*				*Non-adjusted*			
POC	**1.009 (1.000-1.017)**	**0.041**	522.337	Tumor size, mm	**1.202 (1.064-1.358)**	**0.003**	517.436
Sex	1.535 (0.941-2.506)	0.086	523.567				
Age at exposure, years	0.994 (0.955-1.034)	0.754	526.323				
Latency period, years	0.971 (0.935-1.009)	0.139	524.262				
Age at operation, years	0.983 (0.957-1.010)	0.213	524.886				
Thyroid dose, mGy (log)	**1.270 (1.055-1.530)**	**0.012**	519.968				
	*Model 1A*		521.468		*Model 1B*		519.473
Sex	1.393 (0.843-2.301)	0.196		Sex	1.481 (0.899-2.440)	0.196	
Age at exposure, years	1.033 (0.982-1.086)	0.207		Age at exposure, years	0.990 (0.951-1.031)	0.636	
Latency period, years	0.983 (0.945-1.022)	0.395		Latency period, years	0.980 (0.943-1.020)	0.326	
Thyroid dose, mGy (log)	**1.354 (1.061-1.728)**	**0.015**		Tumor size, mm	**1.198 (1.058-1.355)**	**0.004**	
	*Model 2A*		521.884		*Model 2B*		517.589
Sex	1.449 (0.881-2.383)	0.144		Sex	1.449 (0.881-2.383)	0.144	
Age at operation, years	1.002 (0.971-1.034)	0.912		Age at operation, years	1.002 (0.971-1.034)	0.912	
Thyroid dose, mGy (log)	**1.260 (1.013-1.568)**	**0.038**		Tumor size, mm	**1.201 (1.063-1.358)**	**0.003**	
***Models combining POC components and tumor size***							
	*Model 3A*		515.101		*Model 3B*		514.593
Sex	1.395 (0.840-2.315)	0.198		Sex	1.439 (0.870-2.380)	0.157	
Age at exposure, years	1.029 (0.977-1.083)	0.277		Age at operation, years	1.004 (0.972-1.037)	0.819	
Latency period, years	0.989 (0.950-1.029)	0.588		Thyroid dose, mGy (log)	**1.285 (1.026-1.610)**	**0.029**	
Thyroid dose, mGy (log)	**1.365 (1.063-1.752)**	**0.015**		Tumor size, mm	**1.209 (1.069-1.369)**	**0.003**	
Tumor size, mm	**1.200 (1.059-1.359)**	**0.004**					

a Akaike information criterion.

Numbers in bold indicate statistical significance.

Similar approach was applied to analyze extrathyroidal extension (**Table 3**). The increase in the frequency of extrathyroidal extension with increasing POC appeared to be due to thyroid dose but not to any other POC components (non-adjusted models, and Models 1A, 2A). MPTC size effect was also statistically significant (non-adjusted model, and Models 1B and 2B). However, the only valid model in which thyroid dose and tumor size could be combined (Model 3A) demonstrated that thyroid dose effect lost its statistical significance, indicating that independent effect of tumor size was stronger. This, however, does not imply that the effect of thyroid dose on extrathyroidal extension does not exist, rather it could not be confirmed in view of the stronger impact of tumor size.

We also clarified that the reduction in the frequency of tumors with a size greater than the median with increasing POC could be ascribed to the effect of latency period but not to radiation dose (**Table 5**).

**Table 5:** Effects of POC or its components on tumor size (greater than median).

	b (95% CI)	p-value	AIC^a^
*Non-adjusted*			
POC	**0.991 (0.983-0.998)**	**0.017**	641.993
Sex	0.950 (0.608-1.484)	0.820	647.799
Age at exposure, years	**1.039 (1.003-1.076)**	**0.032**	643.192
Latency period, years	**0.965 (0.932-0.999)**	**0.043**	643.708
Age at operation, years	1.001 (0.978-1.025)	0.930	647.842
Thyroid dose, mGy (log)	0.896 (0.762-1.055)	0.188	646.086
	*Model 1*		644.147
Sex	0.917 (0.581-1.445)	0.708	
Age at exposure, years	1.035 (0.993-1.078)	0.105	
Latency period, years	**0.961 (0.927-0.995)**	**0.027**	
Thyroid dose, mGy (log)	0.948 (0.781-1.151)	0.591	
	*Model 2*		649.687
Sex	0.969 (0.618-1.520)	0.893	
Age at operation, years	0.991 (0.965-1.019)	0.535	
Thyroid dose, mGy (log)	0.872 (0.724-1.051)	0.152	

a Akaike information criterion.

Numbers in bold indicate statistical significance.

### Treatment options

In most cases (405 out of 465, 87.1%), total thyroidectomy was performed, and organ-preserving surgery (12.9%), namely hemithyroidectomy and isthmusectomy, were performed in 58 and 2 cases, respectively. MPTCs treated by total thyroidectomy significantly differed from those treated by organ-preserving operation (**Table 6**). They had larger size (OR = 1.225, p = 0.013, and OR = 2.007, p = 0.017 for the size larger than the median), lower frequency of full encapsulation (OR = 0.223, p = 6.91E-07), more frequent dominant papillary growth pattern (OR = 1.807, p = 0.039) and concomitant chronic thyroiditis (OR = 3.365, p = 0.007), and a higher integrative invasiveness score (OR = 3.026, p = 9.00E-06) and its components, such as multifocality (OR = 3.389, p = 0.011), lymphatic/vascular invasion (OR = 2.175, p = 0.042), extrathyroidal extension (OR = 5.555, p = 0.020). Dissection of both central (OR = 2.412, p = 0.020) and lateral (OR = 11.666, p = 0.016) lymph nodes were also performed more frequently in patients undergoing total thyroidectomy.

**Table 6:** Characteristics of the radiogenic papillary thyroid microcarcinomas by thyroid surgery extent.

	Total thyroidectomy, n=405	Organ-preserving, n=60	p-value	OR or HR (95% CI)	p-value
Parameters	number or value (% or IQR or SD)	number or value(% or IQR or SD)	univariate	multivariate^a^	
**Sex**, F/M (%M, F:M ratio; ref=F)	323/82; 20.2%; 1.8:1	44/16; 26.7%; 2.8:1	0.308	0.729 (0.389-1.365)^b^	0.324
**Age at operation**, years	35.3 (30.1-40.2)	34.1 (29.3-36.7)	0.074	1.024 (0.990-1.059)^c^	0.176
**Age at exposure**, years	9.2 (5.0-13.9)	7.4 (3.5-13.2)	0.199	1.034 (0.982-1.089)^c^	0.200
**Latency period**, years	26.1 (22.6-29.2)	26.7 (21.7-28.8)	0.866	1.017 (0.968-1.069)^c^	0.501
**Radiation dose to the thyroid, mGy**	46.8 (29.5-110.7)	46.0 (26.1-113.7)	0.487	1.215 (0.954-1.548)^c^	0.115
**Probability of causation, %**	19.2 (8.7-47.0)	18.7 (7.3-44.3)	0.701	1.000 (0.990-1.011)^d^	0.934
≤ 25%	229 (56.5%)	32 (53.3%)	0.677	1.138 (0.661-1.961)^d^	0.640
> 25 – 50%	86 (21.2%)	15 (25.0%)	0.505	0.899 (0.656-1.233)^d^	0.510
> 50 – 75%	57 (14.1%)	10 (16.7%)	0.559	0.936 (0.732-1.195)^d^	0.594
> 75 – 100%	33 (8.1%)	3 (5.0%)	0.603	1.139 (0.841-1.544)^d^	0.399
**Tumor size**, mm	8 (6-9)	7 (6-8)	0.013	**1.225 (1.044-1.439)**	**0.013**
lesser or equal median	202 (49.9%)	40 (66.7%)	0.018	**0.498 (0.281-0.883)**	**0.017**
greater than median	203 (50.1%)	20 (33.3%)	0.018	**2.007 (1.132-3.559)**	**0.017**
**Full tumor capsule**	55 (13.6%)	25 (41.7%)	1.00E-06	**0.223 (0.124-0.404)**	**6.91E-07**
**Dominant growth pattern**			0.106	**0.704 (0.516-0.962)**^e^	**0.028**
papillary	205 (50.6%)	22 (36.7%)	0.052	**1.807 (1.029-3.171)**	**0.039**
follicular	82 (20.2%)	14 (23.3%)	0.609	0.878 (0.458-1.683)	0.694
solid-trabecular	118 (29.2%)	24 (40.0%)	0.099	0.573 (0.325-1.011)	0.054
**Ki-67 labeling index**, n=92	n=85; 4.6 (2.9-7.2)	n=7; 4.2 (3.2-6.4)	0.854	1.042 (0.814-1.333)	0.745
0 – 5%	48 (56.5%)	4 (57.1%)	1,000	1.002 (0.204-4.915)	0.998
>5 – 10%	31 (36.5%)	3 (42.9%)	0.707	0.746 (0.153-3.653)	0.718
>10%	6 (7.0%)	0	1,000	1.349 (0.315-5.774)	0.686
***BRAF^V600E^*-positive**, n=95	n=88; 60 (68.2%)	n=7; 4 (57.1%)	0.679	0.972 (0.143-6.623)	0.977
**Oncocytic changes**	177 (43.7%)	24 (40.0%)	0.676	1.071 (0.608-1.888)	0.812
**Multifocality**	98 (24.2%)	5 (8.3%)	0.004	**3.389 (1.316-8.725)**	**0.011**
**Lymphatic/vascular invasion**	107 (26.4%)	9 (15.0%)	0.057	**2.175 (1.028-4.600)**	**0.042**
**Extrathyroidal extension** (any)	62 (15.3%)	2 (3.3%)	0.008	**5.555 (1.317-23.430)**	**0.020**
**T category**					
pT1a	398 (98.3%)	60 (100%)	0.602	0.432 (0.020-9.395)	0.593
pT3b	7 (1.7%)	0	0.602	2.314 (0.106-50.308)	0.593
**N category (N1)**	90 (22.2%)	0	2.00E-06	**37.037 (2.268-500.000)**	**0.011**
pN1a	56 (13.8%)	0	4.31E-04	**20.706 (1.264-339.232)**	**0.034**
pN1b	34 (8.4%)	0	0.014	11.425 (0.679-192.354)	0.091
**M category (M1)**	4 (1.0%)	0	1,000	1.723 (0.063-47.224)	0.747
**Invasiveness score**	1 (0-1)	0 (0-0)	3.68E-06	**3.026 (1.857-4.932)**^e^	**9.00E-06**
0	178 (44.0%)	46 (76.7%)	2.00E-06	**0.235 (0.125-0.443)**	**7.00E-06**
1	129 (31.9%)	12 (20.0%)	0.071	1.813 (0.929-3.539)	0.081
2	67 (16.5%)	2 (3.3%)	0.006	**5.915 (1.407-24.861)**	**0.015**
3	26 (6.4%)	0	0.036	9.950 (0.574-172.596)	0.115
4	5 (1.2%)	0	1,000	1.599 (0.067-38.201)	0.772
5	0	0	NA^f^	NA	NA
**Concomitant thyroid cancer**	2 (0.5%)	0	1,000	1.674 (0.040-70.712)	0.787
**Concomitant nodular disease**	110 (27.2%)	10 (16.7%)	0.113	1.715 (0.832-3.537)	0.144
**Concomitant Graves’ disease**	5 (1.2%)	2 (3.3%)	0.225	0.311 (0.058-1.659)	0.171
**Chronic thyroiditis**	115 (28.4%)	6 (10.0%)	0.002	**3.365 (1.396-8.111)**	**0.007**
**LN dissection performed**	182 (44.9%)	10 (6.7%)	1.90E-05	**4.245 (2.0838.651)**	**6.90E-05**
level ≥ 6	123 (30.4%)	9 (15.0%)	0.014	**2.412 (1.149-5.064)**	**0.020**
level 1 – 5	59 (14.6%)	1 (1.7%)	0.003	**11.666 (1.571-86.662)**	**0.016**
**RIT performed**	n=353; 87.2	0	1.18E-44	**781.571 (49.263-inf)**	**2.32E-06**
**RIT cycles**	1 (1-1)	NA	NA	NA	NA
**Cumulative RI activity**, MBq	3964 (2775-4360)	NA	NA	NA	NA
**RIT response**	n=353	NA	NA	NA	NA
RAI-R recurrence *vs* other	3 (0.8%)	NA	NA	NA	NA
excellent *vs* other	332 (94.1%)	NA	NA	NA	NA
**Follow-up**, years	5.3 (2.4-9.1)	3.1 (1.2-7.9)	0.047	1.057 (0.996-1.121)	0.068
**Recurrence**	6 (1.5%)	0	1,000	2.273 (0.100-51.806)^g^	0.607
**Time to recurrence**, yrs	1.2 (1.1-1.6)	NA	NA	NA	NA
**Recurrent metastases**	n=6	0	NA	NA	NA
Dominant growth pattern					
papillary	5 (83.3%)	NA	NA	NA	NA
follicular	1 (16.7%)	NA	NA	NA	NA
solid-trabecular	0	NA	NA	NA	NA
Ki67 labeling index	n=3; 1.2	NA	NA	NA	NA
*BRAF^V600E^*-positive	n=3; 2 (66.7%)	NA	NA	NA	NA
Oncocytic changes	3 (50.0%)	NA	NA	NA	NA
Cystic changes	5 (83.3%)	NA	NA	NA	NA
RIT recurrence response	n=6	NA	NA	NA	NA
RAI-R recurrence *vs* other	3 (50.0%)	NA	NA	NA	NA
excellent *vs* other	3 (50.0%)	NA	NA	NA	NA

a Adjusted for age at operation and sex unless otherwise specified.

b Adjusted for age at operation.

c Adjusted for sex.

d Non-adjusted.

e Polytomous logistic regression.

f Not available.

g The Firth’s-penalized Cox regression.

Numbers in bold indicate statistical significance.

It should also be noted that distant metastases (4 cases, 1.0%) and recurrent metastases (6 cases, 1.5%) were found exclusively in patients who underwent total thyroidectomy (see **Table 6**). Distant metastases were associated with the presence of regional metastases (OR = 8.959, p = 0.016), particularly of the lateral lymph node metastases (OR = 12.745, p = 0.004), a higher integrative invasiveness score (OR = 2.076, p = 0.044), namely the scores “3” (OR = 13.066, p = 0.005) and “4” (OR = 54.188, p = 0.001) (**Supplementary Table 2**). Such patients required more RIT courses (OR = 5.881, p = 0.002) and higher cumulative radioiodine activity (OR = 9.264, p = 0.002). The presence of distant metastases was also associated with the decrease in the frequency of excellent response to RIT (OR = 0.055, p = 0.003), but not with a higher chance of recurrence, including the RAI-R metastases (see **Supplementary Table 2**).

A higher chance of recurrence (6 cases, **Supplementary Table 3**), was associated with more frequent Ki67 LI above 10% (HR = 31.066, p = 0.033) in the primary tumor, more frequent primary central lymph node metastases (HR=7.058, p = 0.018), and the highest in this study invasiveness score of “4” (HR=38.268, p = 0.001). Recurrent tumors could also be linked to the higher number of RIT cycles (HR = 3.403, p = 0.003), less favorable RIT response (HR = 0.258, p = 9.3E-04), higher frequency of RAI-R (OR = 312.985, p = 5.84E-04) and less frequent excellent response to RIT (HR = 0.049, p = 6.76E-04).

**Figure 5:**
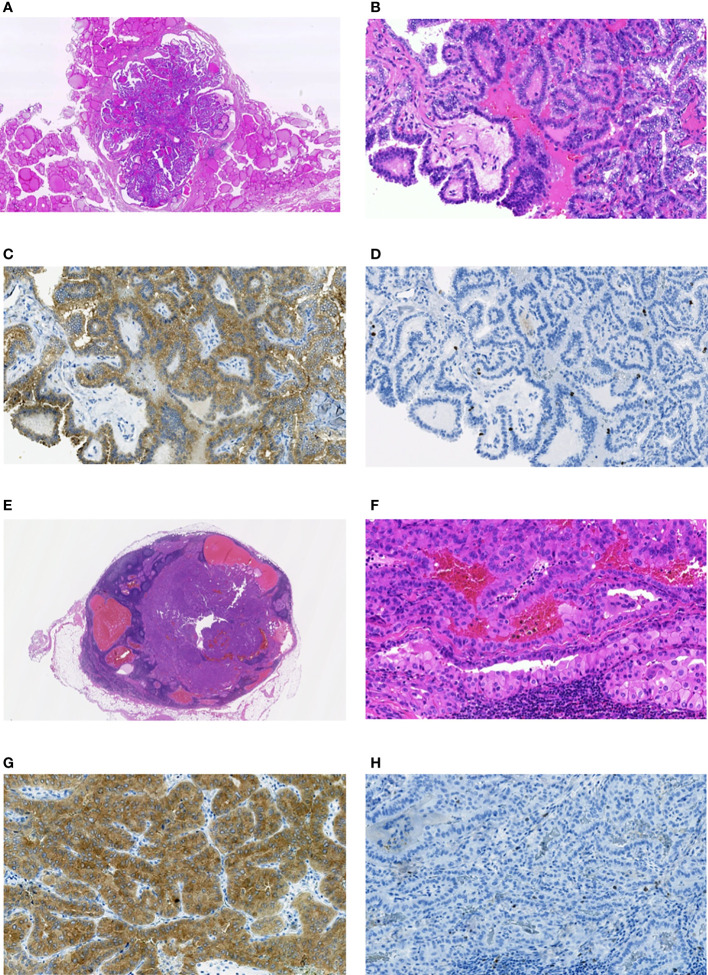
Radiogenic MPTC from male patient aged 11.6 years at the time of the Chornobyl accident and 42 years at the time of the first surgery (estimated (POC=11%), and a RAI-R recurrent metastasis removed 1.6 years after the primary treatment. **(A)** non-encapsulated primary tumor sized 6 mm with the dominant papillary growth pattern, without extrathyroidal extension (pT1aN0M0 according to the 8^th^ Edition of TNM classification), hematoxylin-eosin, 14.5X magnification. **(B)** fragment of the primary tumor with papillary structure, hematoxylin-eosin, 200X magnification. **(C)** primary tumor: positive IHC reaction with anti-BRAF (mutated V600E) antibody, 200X magnification. **(D)** primary tumor: IHC reaction with Ki67 (Clone MIB-1) antibody (Ki67 LI 2.8%), 200X magnification. **(E)** RAI-R recurrent metastasis sized 10 mm with some cystic changes; hematoxylin-eosin, 12.0X magnification. **(F)** fragment of the RAI-R recurrent metastasis: solid-trabecular structure, oncocytic cells, hematoxylin-eosin, 200X magnification. **(G)** fragment of the RAI-R recurrent metastasis: positive IHC reaction with anti-BRAF (mutated V600E) antibody, 200X magnification. **(H)** fragment of the RAI-R recurrent metastasis: IHC reaction with Ki67 (Clone MIB-1) antibody (Ki67 LI 2.0%), 200X magnification.

It is worth noting that among the three recurrent MPTCs for which the additional paraffin sections were available, two primary MPTCs and recurrent metastases were BRAF^V600E^-positive, and metastases were RAI-R (**Figure 5** as an example). This may suggest the mutant BRAF relationship to the mechanism underlying radioiodine refractoriness in radiogenic MPTCs.

## Discussion

This study is the first to assess the effects of the latency period between radiation exposure and MPTC diagnosis, and of the level of the probability of causation of a tumor due to radiation on histopathological and clinical characteristics of MPTCs from internally irradiated patients operated on at the age from 8.8 to 50 years. So far, the only sources of information about radiation-related MPTCs are the report on an increased risk for MPTC in the atomic bomb survivors (7), observations of the growing MPTC frequency with time after the Chornobyl accident (8–11), and the study showing that in young people exposed to Chornobyl radiation at a dose up to 2 Gy, OR/Gy for MPTCs was significantly higher than for tumors larger than 10 mm (OR = 1.20 (95%CI 1.05–1.38), p = 0.004) (30). However, whether radiation exposure may confer higher MPTC aggressiveness, or whether there is a relationship between the latency period and changes in MPTC behavior remained unaddressed. These questions in fact deserve a close attention in view of the increasing frequency of sporadic MPTC worldwide (1–6) and in the individuals exposed to the Chornobyl fallout in childhood (8–11), cautions about treatment approaches to the low-risk PTC in patients previously exposed to radiation (12, 13), and alarming concerns about possible radiation safety violations at nuclear power plants in Ukraine due to Russian military aggression (31).

Our study established that the longer latency period, which is paralleled by the older age of patients at operation, was associated with a decrease in MPTC size without a significant impact on invasive properties (see **Table 2** and **Figure 2**), suggestive that there is no reason to expect worsening of the prognosis after the longer latency. Another favorable prognostic factor, which according to the literature may inhibit tumor progression (32), is the longer latency-related increase in the frequency of concomitant chronic thyroiditis observed in our study.

On the other hand, the longer latency period led to an increase in the frequency of the *BRAF^V600E^* mutation, oncocytic changes, and a decrease in the probability of excellent response to RIT (see **Table 2** and **Figure 2C, F** **)**. The increasing with longer latency period/older patient age frequency of the *BRAF^V600E^* mutation was described in our previous study of radiogenic PTCs, although no special analysis in the context of tumor size was performed in that work (26). A more frequent *BRAF^V600E^* mutation was also found in sporadic PTCs (including MPTCs) in older patients (33–35). Therefore, most likely, it is the older age attained after a longer latency, but not the exposure to Chornobyl fallout that explains the increase in the frequency of BRAF^V600E^-positive cases.

With regard to the decrease in the probability of a complete remission after RIT (see **Table 2** and **Figure 2F**), it is worth noting that there were isolated RAI-R recurrent cases (see **Table 2**) that were BRAF^V600E^-positive in the primary tumor and recurrent metastases (see **Figure 5**). Our data suggest that recurrent tumors may more likely be RAI-refractory and develop from the primary tumors with Ki67 LI greater than 10% (see **Supplementary Table 3**). However, RAI-R recurrent metastases were also detected in our previous study (14) in younger patients with sporadic MPTC. In addition, in the current study of radiogenic MPTCs, no statistically significant relationship between the development of recurrences (including the RAIR-R ones) and the latency period or POC was found (see **Tables 2**, **3** and **Figures 2F**, **3F**). Therefore, the decrease in the probability of a complete remission after RIT in the current MPTC series would unlikely to be due to radiation exposure.

A rather interesting, in our opinion, observation associated with POC level, was an increase in the frequency of lymphatic/vascular invasion (see **Table 4** and **Figure 3D**). We found that among all POC components, the ^131^I thyroid dose was responsible for this association (see **Table 4**). As shown in the previous study (9), the more frequent lymphatic/vascular invasion in the Ukrainian-American cohort members exposed to Chornobyl fallouts in childhood was positively associated with gene fusions (OR = 5.85 (1.43-31.75), p = 0.013), and negatively with point mutations (OR = 0.14 (0.01-0.95), p = 0.044). In earlier works, gene fusions, as compared to point mutations, were associated with a higher POC and a shorter latency period of radiogenic PTC (36, 37). Perhaps, these observations also apply to radiogenic MPTCs, in which a statistically significant decrease in the frequency of the *BRAF^V600E^* mutation was observed with increasing POC (see **Table 2** and **Figure 3C**). It is important that in radiogenic MPTC, the association of lymphatic/vascular invasion frequency with higher POC (and ^131^I radiation dose, see **Table 4**) does not apparently result in its overall higher frequency than in sporadic MPTCs as shown in our study of MPTCs from young patients (14). Perhaps, the effect of radiation, although statistically significant, adds too small excess to the driver oncogene-dependent lymphatic/vascular invasion frequency in the radiogenic MPTCs so that it could not be detected when the radiogenic and sporadic MPTCs groups are directly compared.

The most numerous associations of the indicators of tumor invasiveness (extrathyroidal extension, lymphatic/vascular invasion, lymph node metastases), and the integrative invasiveness score, were with MPTC size (see **Table 3** and **Figure 4D** **).** Tumor size, in turn, was associated with a shorter latency period (see **Tables 2**, **5**). These invasive features are considered to be unfavorable prognostic factors in sporadic MPTCs (38–43), and also were associated with larger MPTC size (44–47), in line with our study. The tendency for tumor size to decline with longer latency again suggests that radiogenic MPTC prognosis would not be expected to be worsening with time after exposure.

As for the thyroid surgery volume, given the presence of radiation exposure in the patients’ histories, total thyroidectomy was performed in most cases (87%), and neck lymph node dissection in nearly a half of the cases. According to our analysis, the major reasons for surgical decision-making, i.e., before the pathological report is available, were the larger tumor size, the likelihood of the absence of full tumor capsule (seen as irregular tumor margins on ultrasound), multifocality, extrathyroidal extension, and lymph node metastases, that could be detected on preoperative imaging. Such a strategy is fully justified and effective *per se*. Of importance also, in cases of hemithyroidectomy (13%), no metastases or disease recurrences were recorded. At the same time, the negative prognostic features occurred in about 20% of cases only (among 465 MPTCs) according to the pathological examination, and they were not associated with a higher chance of recurrence. We therefore suppose that in future clinicians should consider a possibility of more frequent organ-preserving surgeries *versus* total thyroidectomy even for potentially radiogenic MPTCs.

Despite most radiogenic MPTCs in our series were rather indolent, there were several aggressive cases pointing at the need of MPTC stratification into the low-risk and high-risk tumors, as in sporadic MPTC (43, 48, 49). In our opinion, namely such stratification, but not the etiological factor, should be taken into account for decisions on tumor management. Concerning MPTCs, very timely and important changes are expected in the new 5^th^ edition of the WHO Histological Classification of thyroid tumors (50, 51). MPTCs will no longer be just a PTC subtype, but similarly to the tumors of larger size can be considered according to their risk level for personalized treatment protocols.

Summarizing the results, we found no evidence of worsening of the histopathological and clinical features or prognosis of radiogenic MPTCs with the longer latency period or higher POC. The increase in the invasive properties of tumors of a larger size, also well-described in sporadic MPTCs, indicates the need for risk stratification for each MPTC individually regardless of etiology for clinical decision-making.

## Data availability statement

The original contributions presented in the study are included in the article. Further inquiries can be directed to the corresponding author.

## Ethics statement

The studies involving human participants were reviewed and approved by IEM Bioethics Committee (protocols N 22-KE of April 26, 2018, and N 31-KE of February 27, 2020), Chornobyl Tissue Bank (CTB, project N001-2020), Ethics Committee of Nagasaki University (protocol 20130401–7 of July 1, 2021, the latest update). Written informed consent to participate in this study was provided by the participants’ legal guardian/next of kin.

## Author contributions

TB, SC, LZ, NM, MT, SY, and VAS: study design and methodology. TB, SC, LZ, and MB: clinical and pathological data. LZ, TIR, and MI: investigation and formal analysis. SM: thyroid dosimetry. TB and VAS: statistical analysis, data interpretation, and writing of the manuscript. TB, SC, LZ, TIR, NM, MI, MT, MB, SM, SY, and VAS: revision of the manuscript. All authors contributed to the article and approved the submitted version.
