# Supplementary material for: The relationship of the clinicopathological characteristics and treatment results of post-Chornobyl papillary thyroid microcarcinomas with the latency period and radiation exposure
Source: Front Endocrinol (Lausanne). 2022 Dec 14;13:1078258. doi: 10.3389/fendo.2022.1078258 (PMC9796818; doi:10.3389/fendo.2022.1078258)
Supplement: Supplementary file 1 [file Table_1.docx]

**Supplementary Table 1** Characteristics of the seven radiogenic papillary thyroid microcarcinomas with gross extrathyroidal extension (pT3b category)

| **Parameters** | **pT3b category, n=7** |  | **OR or HR (95%CI)** | **p-value** |
| --- | --- | --- | --- | --- |
|  | **number or value** |  | **multivariate^a^** | |
|  | **(% or IQR or SD)** |  |  |  |
| **Sex**, F/M; %M; F:M ratio (ref=F) | 5/2; 28.6%; 2.5:1 |  | 1.554 (0.296-8.149)^b^ | 0.602 |
| **Age at operation**, years | 35.4 (33.7-38.3) |  | 1.029 (0.930-1.140)^c^ | 0.578 |
| **Age at exposure**, years | 5.6 (2.8-9.2) |  | 0.901 (0.774-1.048)^c^ | 0.175 |
| **Latency period**, years | 31.8 (27.8-32.0) |  | **1.318 (1.020-1.703)**^c^ | **0.034** |
| **Radiation dose to the thyroid**, mGy | 116.0 (105.7-168.1) |  | 1.351 (0.772-2.363)^c^ | 0.292 |
| **Probability of causation**, % | 49.7 (42.4-52.1) |  | 1.021 (0.995-1.047)^d^ | 0.122 |
| ≤ 25% | 1 (14.3%) |  | 0.127 (0.015-1.063)^d^ | 0.057 |
| > 25 – 50% | 3 (42.9%) |  | 1.660 (0.779-3.538)^d^ | 0.189 |
| > 50 – 75% | 3 (42.9%) |  | **1.665 (1.003-2.764)**^d^ | **0.049** |
| > 75 – 100% | 0 |  | NA^e^ | NA |
| **Tumor size**, mm | 7.0 (7-9) |  | 1.092 (0.712-1.675) | 0.686 |
| lesser or equal median | 4 (57.1%) |  | 1.214 (0.268-5.500) | 0.802 |
| greater than median | 3 (42.9%) |  | 0.824 (0.182-3.734) | 0.802 |
| **Full tumor capsule** | 0 |  | NA |  |
| **Dominant growth pattern** |  |  | **3.000 (1.041-8.644)** | **0.042** |
| papillary | 0 |  | NA |  |
| follicular | 3 (42.9%) |  | 2.968 (0.648-13.590) | 0.161 |
| solid-trabecular | 4 (57.1%) |  | 3.119 (0.676-14.400) | 0.145 |
| **Ki-67 labeling index** | NA |  | NA | NA |
| 0 – 5% | NA |  | NA | NA |
| > 5 – 10% | NA |  | NA | NA |
| > 10% | NA |  | NA | NA |
| **BRAF^V600E^-positive** | NA |  | NA | NA |
| **Oncocytic changes** | 2 (28.6%) |  | 0.473 (0.089-2.520) | 0.380 |
| **Multifocality** | 1 (14.3%) |  | 0.558 (0.066-4.725) | 0.593 |
| **Lymphatic/vascular invasion** | 3 (42.9%) |  | 2.293 (0.501-10.500) | 0.285 |
| **Extrathyroidal extension** | 7 (100%) |  | NA | NA |
| **T category** |  |  | NA | NA |
| pT1a | 0 |  | NA | NA |
| pT3b | 7 (100%) |  | NA | NA |
| **N category (N1)** | 5 (71.5%) |  | **10.807 (2.040-57.244)** | **0.005** |
| N1a | 3 (42.9%) |  | **5.816 (1.252-27.016)** | **0.025** |
| N1b | 2 (28.6%) |  | 4.940 (0.898-27.158) | 0.066 |
| **M category (M1)** | 0 |  | NA | NA |
| **Invasiveness score** | 2 (2-3) |  | **3.236 (1.663-6.299)** | **5.47E-04** |
| 0 | 0 |  | NA | NA |
| 1 | 1 (14.3%) |  | 0.381 (0.045-3.209) | 0.375 |
| 2 | 3 (42.9%) |  | 4.385 (0.954-20.148) | 0.057 |
| 3 | 3 (42.9%) |  | **14.813 (2.942-74.576)** | **0.001** |
| 4 | 0 |  | NA | NA |
| 5 | 0 |  | NA | NA |
| **Concomitant thyroid cancer** | 0 |  | NA | NA |
| **Concomitant nodular disease** | 1 (14.3%) |  | 0.439 (0.051-3.793) | 0.455 |
| **Concomitant Graves' disease** | 0 |  | NA | NA |
| **Chronic thyroiditis** | 1 (14.3%) |  | 0.478 (0.055-4.151) | 0.504 |
| **Thyroid surgery** |  |  |  |  |
| total thyroidectomy | 7 (100%) |  | NA | NA |
| organ-preserving operation | 0 |  | NA | NA |
| **LN dissection performed** | 6 (85.7%) |  | **8.636 (1.028-72.541)** | **0.047** |
| level ≥ 6 | 4 (66.7%) |  | 3.402 (0.748-15.467) | 0.113 |
| level 1 – 5 | 2 (33.3%) |  | 2.757 (0.507-15.000) | 0.241 |
| **RIT performed** | 6 (85.7%) |  | 1.961 (0.231-16.628) | 0.537 |
| **RIT cycles**, n=6 | 1 (1-1) |  | 1.301 (0.444-3.808) | 0.632 |
| **Cumulative RI activity, MBq** | 4360 (4360-4360) |  | 1.784 (0.772-4.123) | 0.176 |
| **RIT response**, n=6 |  |  | 0.668 (0.269-1.654) | 0.383 |
| RAI-R recurrence *vs* other | 0 |  | NA | NA |
| excellent *vs* other | 5 (83.3%) |  | 0.303 (0.031-2.931) | 0.302 |
| **Follow-up, years** | 3.6 (1.9-5.8) |  | 0.924 (0.763-1.120) | 0.420 |
| **Recurrence** | 0 |  | NA | NA |
| **Time to recurrence**, years | NA |  | NA | NA |
| **Recurrent metastases** | 0 |  | NA | NA |

^a^ Adjusted for age at operation and sex unless otherwise specified

^b^ Adjusted for age at operation

^c^ Adjusted for sex

^d^ Non-adjusted

^e^ Not available

Numbers in bold indicate statistical significance

**Supplementary Table 2** Characteristics of the four radiogenic papillary thyroid microcarcinomas with distant metastases to the lung

|  | **M1, n=4** |  | **OR or HR (95%CI)** | **p-value** |
| --- | --- | --- | --- | --- |
| **Parameters** | **number or value** |  | **multivariate^a^** | |
|  | **(% or IQR or SD)** |  |  |  |
| **Sex F/M (%M, F:M ratio; ref=F)** | 2/2; 50.0; 1:1 |  | 3.497 (0.619-19.750)^b^ | 0.156 |
| **Age at operation**, years | 29.2 (24.7-36.0) |  | 0.953 (0.867-1.047)^c^ | 0.315 |
| **Age at exposure**, years | 2.7 (1.0-10.6) |  | 0.903 (0.764-1.068)^c^ | 0.235 |
| **Period of latency**, years | 23.2 (22.7-26.5) |  | 0.979 (0.846-1.133)^c^ | 0.779 |
| **Radiation dose to the thyroid**, mGy | 93.0 (57.9-265.5) |  | 1.396 (0.795-2.451)^c^ | 0.246 |
| **Probability of causation**, % | 48.6 (27.6-65.9) |  | 1.024 (0.993-1.055)^d^ | 0.133 |
| ≤ 25% | 1 (25.0%) |  | 0.332 (0.048-2.274)^d^ | 0.261 |
| > 25 – 50% | 1 (25.0%%) |  | 1.242 (0.472-3.264)^d^ | 0.661 |
| > 50 – 75% | 1 (25.0%) |  | 1.366 (0.715-2.609)^d^ | 0.345 |
| > 75 – 100% | 1 (25.0%) |  | 1.506 (0.922-2.461)^d^ | 0.102 |
| **Tumor size**, mm | 8.0 (5.5-9.0) |  | 0.927 (0.580-1.481) | 0.751 |
| lesser or equal median | 3 (75.0%) |  | 0.401 (0.069-2.342) | 0.310 |
| greater than median | 1 (25.0%) |  | 2.492 (0.427-14.539) | 0.310 |
| **Full tumor capsule** | 0 |  | 0.489 (0.031-7.594) | 0.609 |
| **Dominant growth pattern** |  |  | 1.862 (0.699-4.961) | 0.214 |
| papillary | 1 (25.0%) |  | 0.415 (0.069-2.490) | 0.336 |
| follicular | 1 (25.0%) |  | 1.365 (0.0223-8.334) | 0.736 |
| solid-trabecular | 2 (50.0%) |  | 2.391 (0.552-15.547) | 0.207 |
| **Ki-67 labeling index**, n=2 | 5.6 (2.4-8.7) |  | 1.077 (0.835-1.388) | 0.568 |
| 0 – 5% | 1 (50.0%) |  | 0.752 (0.092-6.162) | 0.791 |
| >5 – 10% | 1 (50.0%) |  | 1.719 (0.210-14.040) | 0.613 |
| >10% | 0 |  | 2.421 (0.097-60.125) | 0.59 |
| **BRAF^V600E^-positive**, n=2 | 1 (50.0%) |  | 0.720 (0.057-9.039) | 0.799 |
| **Oncocytic changes** | 1 (25.0%) |  | 0.686 (0.107-4.392) | 0.690 |
| **Multifocality** | 1 (25.0%) |  | 1.714 (0.278-10.561) | 0.561 |
| **Lymphatic/vascular invasion** | 2 (50.0%) |  | 2.549 (0.490-13.263) | 0.266 |
| **Extrathyroidal extension** | 1 (25.0%) |  | 2.371 (0.387-14.522) | 0.350 |
| **T category** |  |  | 1.503 (0.662-3.410) | 0.330 |
| pT1a | 4 (100%) |  | 0.196 (0.007-5.204) | 0.330 |
| pT3b | 0 |  | 5.099 (0.192-135.270) | 0.330 |
| **N category (N1)** | 3 (75.0%) |  | **8.959 (1.499-53.537)** | **0.016** |
| pN1a | 1 (25.0%) |  | 2.700 (0.436-16.720) | 0.286 |
| pN1b | 2 (50.0%) |  | **12.745 (2.231-72.827)** | **0.004** |
| **M category (M1)** | 4 (100%) |  | NA^e^ | NA |
| **Invasiveness score** | 3.0 (2-3.5) |  | **2.076 (1.018-4.231)** | **0.044** |
| 0 | 0 |  | 0.118 (0.008-1.718) | 0.118 |
| 1 | 1 (25.0%) |  | 1.098 (0.181-6.642) | 0.919 |
| 2 | 0 |  | 0.622 (0.042-9.252) | 0.730 |
| 3 | 2 (50.0%) |  | **13.066 (2.187-78.067)** | **0.005** |
| 4 | 1 (25.0%) |  | **54.188(5.003-586.857)** | **0.001** |
| 5 | 0 |  | NA | NA |
| **Concomitant thyroid cancer** | 0 |  | 38.733 (0.711-inf) | 0.073 |
| **Concomitant nodular disease** | 1 (25.0%) |  | 1.696 (0.2638-10.734) | 0.575 |
| **Concomitant Graves' disease** | 0 |  | 10.413 (0.382-283.503) | 0.165 |
| **Chronic thyroiditis** | 0 |  | 0.411 (0.025-6.669) | 0.532 |
| **Thyroid surgery** |  |  |  |  |
| total thyroidectomy | 4 (100%) |  | 1.516 (0.097-23.774) | 0.767 |
| organ-preserving operation | 0 |  | 0.660 (0.042-10.309) | 0.767 |
| **LN dissection performed** | 4 (100%) |  | 11.910 (0.818-173.404) | 0.070 |
| level ≥ 6 | 2 (50.0%) |  | 2.750 (0.526-14.372) | 0.231 |
| level 1 – 5 | 2 (50.0%) |  | 5.245 (0.964-28.530) | 0.055 |
| **RIT performed** | 4 (100%) |  | 3.293 (0.230-47.037) | 0.380 |
| **RIT cycles** | 2.5 |  | **5.881 (1.879-18.406)** | **0.002** |
| **Cumulative RI activity**, MBq | 11895 (7432-17886) |  | **9.264 (2.242-38.286)** | **0.002** |
| **RIT response** | n=4 |  | **0.429 (0.215-0.855)** | **0.016** |
| RAI-R recurrence *vs* other | 0 |  | 6.630 (0.140-314.757) | 0.337 |
| excellent *vs* other | 2 |  | **0.055 (0.008-0.381)** | **0.003** |
| **Follow-up**, years | 6.2 (1.8-11.1) |  | 0.957 (0.812-1.128) | 0.600 |
| **Recurrence** | 0 |  | 4.469 (0.170-117.594) | 0.370 |
| **Time to recurrence**, years | NA |  | NA | NA |
| **Recurrent metastases** | 0 |  | NA | NA |

^a^ Adjusted for age at operation and sex unless otherwise specified

^b^ Adjusted for age at operation

^c^ Adjusted for sex

^d^ Non-adjusted

^e^ Not available

Numbers in bold indicate statistical significance

**Supplementary Table 3** Characteristics of the six recurrent radiogenic papillary thyroid microcarcinomas

|  | **Recurrent PTCs, n=6** |  | **HR (95%CI)** | **p-value** |
| --- | --- | --- | --- | --- |
| **Parameters** | **number or value** |  | **multivariate^a^** | |
|  | **(% or IQR or SD)** |  |  |  |
| **Sex**, F/M; %M; F:M ratio (ref=F) | 3/3; 50.0%; 1:1 |  | 3.878 (0.771-19.506)^b^ | 0.100 |
| **Age at operation**, years | 31.8 (27.1-42.5) |  | 1.027 (0.928-1.135)^c^ | 0.609 |
| **Age at exposure**, years | 8.6 (3.3-11.6) |  | 1.000 (0.864-1.158)^c^ | 0.996 |
| **Latency period**, years | 24.3 (21.8-30.1) |  | 1.069 (0.904-1.264)^c^ | 0.438 |
| **Radiation dose to the thyroid**, mGy | 41.5 (32.1-78.3) |  | 0.821 (0.449-1.502)^c^ | 0.522 |
| **Probability of causation**, % | 17.2 (11.0-39.2) |  | 0.993 (0.959-1.027)^d^ | 0.677 |
| ≤ 25% | 4 (66.7%) |  | 1.613 (0.295-8.808)^d^ | 0.581 |
| > 25 – 50% | 1 (16.7%) |  | 0.872 (0.297-2.555)^d^ | 0.802 |
| > 50 – 75% | 1 (16.7%) |  | 1.048 (0.511-2.146)^d^ | 0.899 |
| > 75 – 100% | 0 |  | 0.455 (0.021-9.897)^d^ | 0.616 |
| **Tumor size**, mm | 9.0 (6-10) |  | 1.348 (0.814-2.232) | 0.246 |
| lesser or equal median | 3 (50.0%) |  | 0.479 (0.087-2.632) | 0.397 |
| greater than median | 3 (50.0%) |  | 2.089 (0.380-11.472) | 0.397 |
| **Full tumor capsule** | 1 (16.7%) |  | 0.332 (0.003-2.840) | 0.492 |
| **Dominant growth pattern** |  |  | 0.208 (0.003-0.877) | 0.142 |
| papillary | 6 (100%) |  | 11.643 (1.373-1520.035) | 0.115 |
| follicular | 0 |  | 0.307 (0.002-2.627) | 0.451 |
| solid-trabecular | 0 |  | 0.196 (0.001-1.703) | 0.304 |
| **Ki-67 labeling index**, n=3 | 2.8 (1.9-7.9) |  | 1.142 (0.778-1.674) | 0.498 |
| 0 – 5% | 2 (66.7%) |  | 0.943 (0.118-10.663) | 0.962 |
| >5 – 10% | 0 |  | 0.281 (0.002-2.960) | 0.473 |
| >10% | 1 (33.3%) |  | **31.066 (1.906-589.118)** | **0.033** |
| **BRAF^V600E^-positive**, n=3 | 2 (66.7%) |  | 0.438 (0.032-6.046) | 0.538 |
| **Oncocytic changes** | 3 (50.0%) |  | 1.419 (0.261-7.709) | 0.685 |
| **Multifocality** | 2 (33.3%) |  | 1.857 (0.337-10.240) | 0.477 |
| **Lymphatic/vascular invasion** | 2 (33.3%) |  | 1.339 (0.242-7.409) | 0.738 |
| **Extrathyroidal extension** | 2 (33.3%) |  | 2.797 (0.503-15.569) | 0.240 |
| **T category** |  |  |  |  |
| pT1a | 6 (100%) |  | 4.967 (0.038-42.583) | 0.324 |
| pT3b | 0 |  | 0.201 (0.023-26.379) | 0.324 |
| **N category (N1)** | 3 (50.0%) |  | 3.902 (0.774-19.676) | 0.099 |
| pN1a | 3 (50.0%) |  | **7.058 (1.466-34.069)** | **0.018** |
| pN1b | 0 |  | 0.883 (0.007-7.640) | 0.938 |
| **M category (M1)** | 0 |  | 6.350 (0.047-57.398) | 0.258 |
| **Invasiveness score** | 1 (1-2) |  | 1.710 (0.963-3.388) | 0.124 |
| 0 | 1 (16.7%) |  | 0.217 (0.025-1.873) | 0.165 |
| 1 | 3 (50.0%) |  | 2.517 (0.504-12.562) | 0.260 |
| 2 | 1 (16.7%) |  | 1.130 (0.131-9.716) | 0.911 |
| 3 | 0 |  | 0.841 (0.006-7.575) | 0.914 |
| 4 | 1 (16.7%) |  | **38.268 (3.636-244.856)** | **0.001** |
| 5 | 0 |  | NA^e^ | NA |
| **Concomitant thyroid cancer** | 0 |  | **37.572 (0.275-410.785)** | **0.042** |
| **Concomitant nodular disease** | 3 (50.0%) |  | 3.878 (0.731-20.563) | 0.111 |
| **Concomitant Graves’ disease** | 0 |  | 6.226 (0.046-65.177) | 0.292 |
| **Chronic thyroiditis** | 1 (16.7%) |  | 0.770 (0.085-7.021) | 0.817 |
| **Thyroid surgery** |  |  |  |  |
| total thyroidectomy | 6 (100%) |  | 1.785 (0.211-232.791) | 0.716 |
| organ-preserving operation | 0 |  | 0.560 (0.004-4.744) | 0.716 |
| **LN dissection performed** | 5 (83.3%) |  | 6.478 (0.751-55.857) | 0.089 |
| level ≥ 6 | 4 (66.7%) |  | 5.485 (0.989-30.418) | 0.051 |
| level 1 – 5 | 1 (16.7%) |  | 0.994 (0.113-8.742) | 0.996 |
| **RIT performed** | 6 (100%) |  | 3.464 (0.407-452.392) | 0.427 |
| **RIT cycles** | 2 (2-2) |  | **3.403 (1.584-8.213)** | **0.003** |
| **Cumulative RI activity**, MBq | 6958 (4360-8720) |  | **4.217 (1.428-11.182)** | **0.007** |
| **RIT response** |  |  | **0.258 (0.095-0.502)** | **9.30E-04** |
| RAI-R recurrence *vs* other | 3 (50.0%) |  | **312.985 (20.090-17900.840)** | **5.84E-04** |
| excellent *vs* other | 3 (50.0%) |  | **0.049 (0.009-0.268)** | **6.76E-04** |
| **Follow-up**, years | 7.9 (4.7-11.5) |  | 0.979 (0.802-1.196) | 0.839 |
| **Recurrence** | n=6 |  | NA | NA |
| **Time to recurrence**, years | 1.2 (1.1-1.6) |  | 0.853 (0.407-1.135) | 0.444 |
| **Recurrent metastases**, n=6 |  |  | NA | NA |
| Dominant growth pattern |  |  |  |  |
| papillary | 5 (83.3%) |  | 0.017 (0.000-1.105) | 0.171 |
| follicular | 1 (16.7%) |  | 57.817 (0.905-inf) | 0.171 |
| solid-trabecular | 0 |  | NA | NA |
| Ki67 labeling index | n=3; 1.2 |  | NA | NA |
| BRAF^V600E^-positive | n=3; 2 (66.7%) |  | NA | NA |
| Oncocytic changes | 3 (50.0%) |  | 0.806 (0.074-63717) | 0.858 |
| Cystic changes | 5 (83.3%) |  | 3.915 (0.253-556.946) | 0.475 |
| RIT recurrence response | n=6 |  | 2.288 (0.459-122.901) | 0.452 |
| RAI-R recurrence *vs* other | 3 (50.0%) |  | 0.036 (0.000-22.520) | 0.452 |
| excellent *vs* other | 3 (50.0%) |  | 27.417 (0.044-inf) | 0.452 |

^a^ Adjusted for age at operation and sex unless otherwise specified

^b^ Adjusted for age at operation

^c^ Adjusted for sex

^d^ Non-adjusted

^e^ Not available

Numbers in bold indicate statistical significance
